# Invasive and non-invasive electrodes for successful drug and gene delivery in electroporation-based treatments

**DOI:** 10.3389/fbioe.2022.1094968

**Published:** 2023-01-16

**Authors:** Veronika Malyško-Ptašinskė, Gediminas Staigvila, Vitalij Novickij

**Affiliations:** ^1^ Faculty of Electronics, Vilnius Gediminas Technical University, Vilnius, Lithuania; ^2^ Department of Immunology, State Research Institute Centre of Innovative Medicine, Vilnius, Lithuania

**Keywords:** electrodes, electroporation, spatial electric field distribution, tumors, electrical tissue properties

## Abstract

Electroporation is an effective physical method for irreversible or reversible permeabilization of plasma membranes of biological cells and is typically used for tissue ablation or targeted drug/DNA delivery into living cells. In the context of cancer treatment, full recovery from an electroporation-based procedure is frequently dependent on the spatial distribution/homogeneity of the electric field in the tissue; therefore, the structure of electrodes/applicators plays an important role. This review focuses on the analysis of electrodes and *in silico* models used for electroporation in cancer treatment and gene therapy. We have reviewed various invasive and non-invasive electrodes; analyzed the spatial electric field distribution using finite element method analysis; evaluated parametric compatibility, and the pros and cons of application; and summarized options for improvement. Additionally, this review highlights the importance of tissue bioimpedance for accurate treatment planning using numerical modeling and the effects of pulse frequency on tissue conductivity and relative permittivity values.

## 1 Introduction

Electroporation is a phenomenon in which the cell plasma membrane is permeabilized by the application of short, high-intensity electric field pulses. The increased permeabilization of the cell membrane is related to the formation of transient aqueous pores, creating pathways for drugs or DNA molecules to enter the cell (Freeman et al., 1994; Du et al., 2018; Bö et al., 2020). However, in order to create these pores, the transmembrane potential of the cell must exceed the electroporation threshold (Kinosita and Tsong, 1977). Thus, depending on the PEF parameters (pulse duration, strength, repetition frequency, etc.), different cell responses to the treatment could be triggered (Szlasa et al., 2021; Vižintin et al., 2021). In the case of reversible electroporation (RE), after a specific resealing time, membrane integrity is restored and the cell survives. RE can be used in electrochemotherapy (ECT), which is a combination of chemotherapy and electroporation, resulting in a highly effective method for cancer treatment. Additionally, electroporation can be used for controlled electro-transfer of DNA, known as gene electro-transfer (GET) or electro-transfection. However, if the intensity of the PEF is further increased, it may lead to irreversible electroporation (IRE) and consequently cell death, resulting in tissue ablation (Sersa et al., 2008a; Korohoda et al., 2013; Calvet and Mir, 2016; Cemazar and Sersa, 2019). Therefore, depending on the purpose, the desired outcome can be achieved and successfully controlled by modulating the PEF parameters. In clinical settings, in order to predict the effect of electroporation on various tissues and ensure precise electrode positioning, a wide spectrum of techniques is employed (Neal II et al., 2012), including magnetic resonance imaging (Figini et al., 2017; Boc et al., 2018); however, numerical modeling is the most popular method for treatment planning (Kranjc et al., 2017; Asadi et al., 2019). Numerical models can serve as an aid for accurate prediction of electroporation outcome and better pretreatment planning by simulation of the spatial electric field distribution (Miklavčič et al., 1998; Pavšelj and Miklavčič, 2008).

**FIGURE 1:**
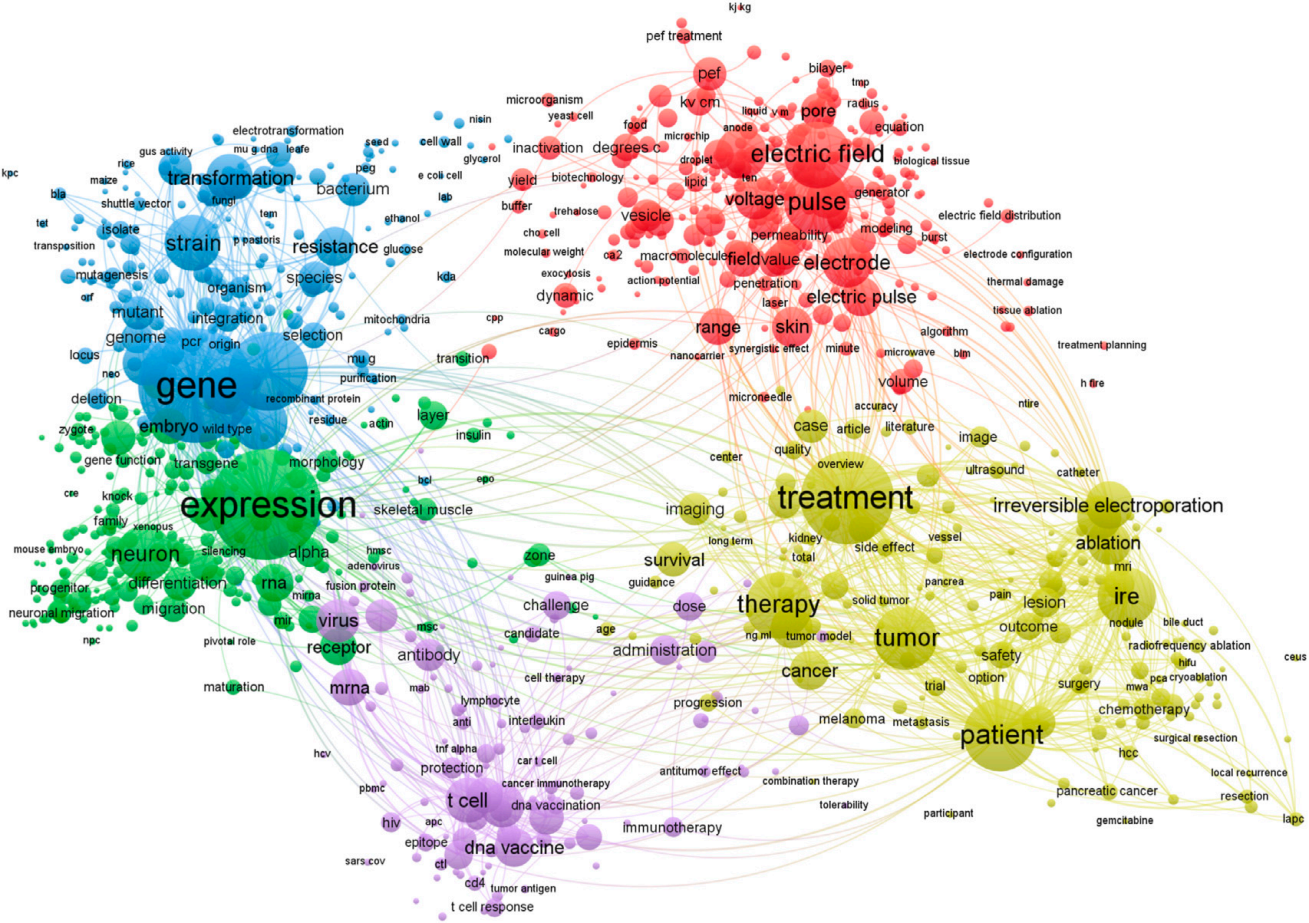
Most common keywords used in electroporation studies. Visualized using VOSviewer software, version 1.6.18 (Leiden University) (VOSviewer - Visualizing scientific landscapes, 2022). A filter of at least 30 minimum occurrences of the keywords has been used. Bigger circle size indicates higher rate of occurrence of a specific keyword.

As a result, electroporation-based technologies and applications are an interdisciplinary field involving research in electronics and electrical engineering, biophysics, biomedicine, microbiology, and food technology. Based on Clarivate Analytics Web of Science, there are 17,179 papers featuring the keyword “electroporation” (Access date: 2022–03-01). A visual map of the most common keywords in electroporation papers is shown in Figure 1.

It can be seen that the applied aspects of electroporation dominate the field, although there is an inevitable interconnectivity with the electric field, electrodes, voltage, and pulse characterization in order to ensure repeatability of the research and protocols (arrangement of red circles).

Generally, electrical pulses are delivered using a high-power generator (electroporator) and electrodes (applicator), where the electrodes transfer the energy of the pulse to the biological tissue. As a result, various models and prototypes of electrodes for electroporation-based treatments are constantly being proposed (Dermol-Černe et al., 2020). Each electrode configuration is unique and frequently limited to a specific target tissue or designated area of application. In this work, we provide an overview of various electrode structures and describe the pros and cons, suitable protocols, and applications. To support better understanding and enable adequate comparison, we also performed the simulation of spatial electric field distribution using the finite element method (FEM). The modeling and comparative analysis of various electrode types are presented in Section 2 of the review. Also, the summary of pulse parameters and protocols used with different electrode types is presented in Section 3.

Nevertheless, the accuracy of any model is determined by the extent of the approximations included. For better pretreatment planning through numerical modeling, inclusion of the peculiarities of the treated object structure is critical. The composition of biological tissues is heterogeneous; i.e., it consists of various layers and structures with specific electrical properties and, thus, different responses to PEF. In order to analyze the outcome of electrical pulsing on tissues, its composition and dielectric properties (specific conductivity and relative permittivity) have to be considered. Therefore, this review also summarizes the frequency-dependent dielectric properties of various healthy and cancerous tissues.

## 2 Mammalian tissue electroporation

**FIGURE 2:**
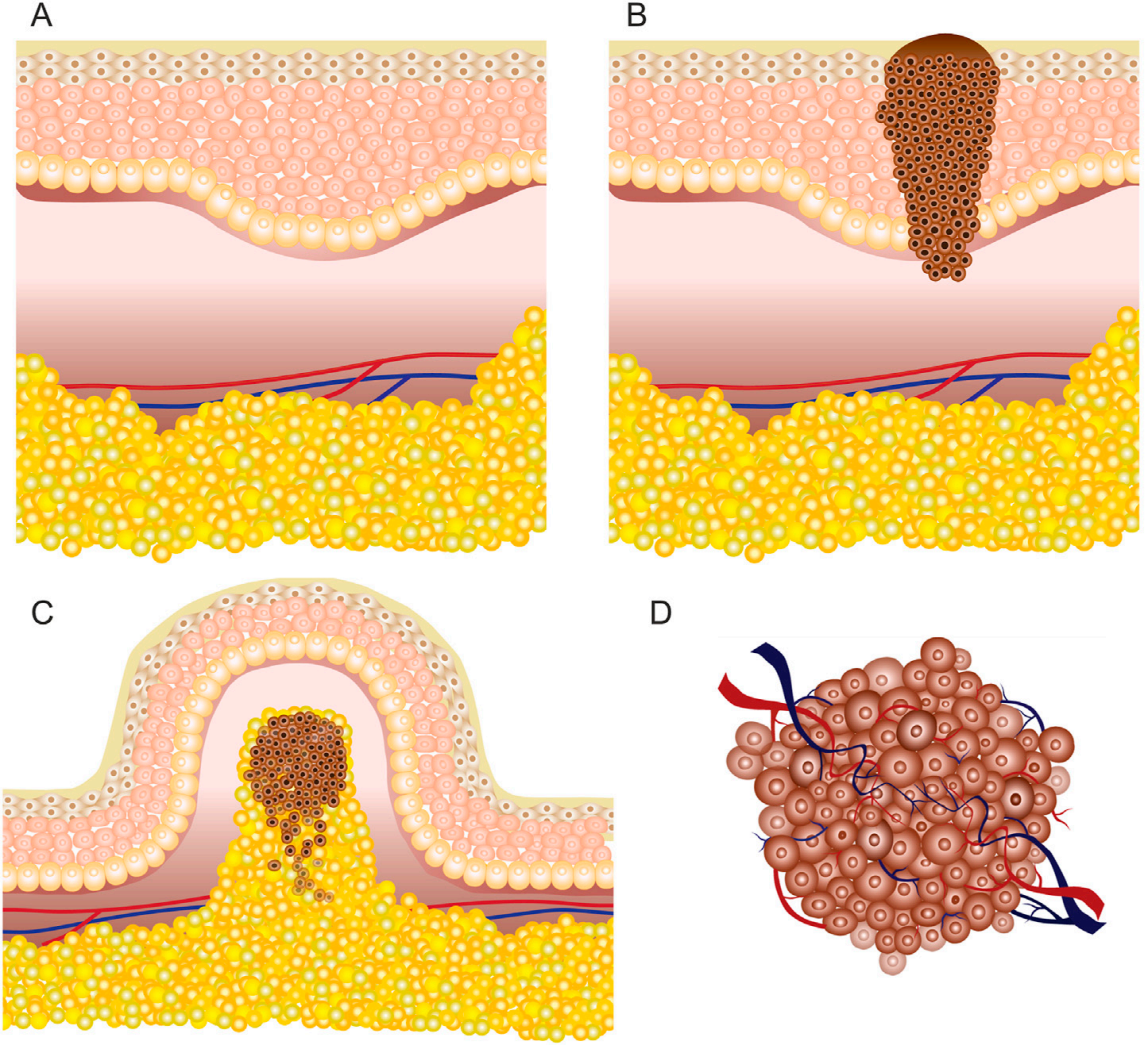
Skin illustration: **(A)** healthy tissue; **(B)** cutaneous tumor (melanoma); **(C)** superficial or exophytic tumor; **(D)** deep-seated tumor.

By *in vivo* and clinical electroporation procedures, various types of tumors can be treated (Li, 2008), which may be grouped simply as cutaneous (located on the skin) or subcutaneous (located under the skin in the subcutaneous tissues) lesions (Figure 2). According to available research, GET, ECT, and IRE are applicable for both deep-seated targets such as tumors/lesions located in muscles and superficial targets located directly on the skin or under the skin (Matthiessen et al., 2012; Heller and Heller, 2015). Consequently, each approach requires specific applicators and their precise positioning, taking into account the location of the target tissue (Zupanic et al., 2012).

According to the European Standard Operating Procedures of Electrochemotherapy (ESOPE) for subcutaneous tumors, pulsed electric field must be generated in deeper tissues; hence, invasive electrodes are required. In contrast, non-invasive electrodes are frequently used for cutaneous targets and have limited applicability for deep-seated tumors (Cvetkoska et al., 2020). Electroporation-based treatment success depends on the coverage of the tumor tissue by sufficiently high local electric field, requiring good contact area between the tissue and the electrodes (Čorović et al., 2008; Sachdev et al., 2022). Simulation of the electric field distribution is usually performed using the FEM analysis (Pintar et al., 2018). However, for accurate prediction, the electrical parameters of the tissue should be known and the heterogeneity should be taken into account, especially for treatment of superficial tumors (Figure 2C) due to high heterogeneity of the skin.

The skin has several functions, including protection of internal organs from environmental influence (Monteiro-riviere and Riviere, 1999; Hayes et al., 2022). A thorough understanding of the skin structure and its electrical properties is crucial to make subcutaneous tumors permeable. Basically, the skin consists of the stratum corneum, epidermis, dermis, hypodermis, fat (subcutaneous adipose tissue), and muscle tissue under the hypodermis (Huclova et al., 2012; Ventrelli et al., 2015). The outer layer (stratum corneum) is mostly composed of dead skin cells and is the thinnest; however, it has the highest resistivity. As a result, the skin is considered a barrier for successful electroporation applications when non-invasive electrodes are employed. The epidermis and dermis are located beneath the stratum corneum and have much lower resistance; therefore, there is a considerable voltage drop across the stratum corneum (Alkilani et al., 2015; Lu et al., 2018). However, once the stratum corneum is permeabilized by the formation of local transport regions (Gelker et al., 2018), deeper layers of the skin can be affected.

However, for deep-seated tumors (Figure 2D) such as tumors of the liver or pancreas, either invasive electrodes are used percutaneously or the treatment is performed during an open surgery (Granata et al., 2021); therefore, the skin has little to no effect on the electroporation procedure. Nevertheless, in most cases with deep-seated tumors, the tumors are intact with healthy organs (Edhemovic et al., 2014) or the tumors are encapsulated into the organs (Ghossein, 2010) and surrounded by large blood vessels (Djokic et al., 2018a). The complexity of such tumor composition influences the inhomogeneity of the target tissue, which may result in non-uniform treatment. As a consequence, the electrical properties of such tumors and tissues in vicinity may vary and, therefore, must be taken into account.

Tissue electrical properties can be described by the concept of bioimpedance, which is a frequency-dependent parameter specific to tissue composition, including water content (Chumlea and Guo, 1994). Biological tissue is considered neither a good conductor nor an insulator but rather something in between that allows the flow of a certain amount of current. This is due to the influence of aqueous, for instance the muscle, and non-aqueous components, such as bone or fat structures. In the low- and high-frequency range, the current density vectors vary, and the bioimpedance decreases in the high-frequency range, enabling a more homogeneous treatment (Raja et al., 2006). Therefore, the conductivity and relative permittivity changes in the tissue and their dependence on the applied pulse frequency should always be taken into account (Miklavčič et al., 2006; Zhang and He, 2010).

Thus, the tissue electrical properties are characterized by its specific conductivity *σ* and relative permittivity ε_r_. It is known that the increase in electric conductivity is related to the formation of local transport regions after the application of electric pulses (Pliquett et al., 1998). Hence, conductivity is the ability of aqueous solutions to transfer electric charge. Simultaneously, the ability of a material to be polarized is characterized by relative permittivity. Consequently, these properties are vital for numerical modeling of the tissue. According to previous studies, the value of conductivity may exhibit a significant increase with the increase in pulse repetition frequency when pulses are applied in bursts with repetition frequency above 100 kHz (de Santis et al., 2015; Weinert and Ramos, 2021), while an opposite dependence is observed for relative permittivity (Valdastri et al., 2004; Peyman et al., 2005). A summary of various tissue conductivities and relative permittivities for different frequency ranges is presented in Supplementary Table S1.

In order to reduce the complexity of numerical models and simplify the calculations, conductivity and relative permittivity may be considered as constant values for low or high PEF frequency ranges, while it should be understood that both parameters are dependent on the applied burst frequency.

Additionally, each electroporation procedure (IRE or RE) requires different pulse parameters and a specific field strength (Čorović et al., 2012; Forjanic et al., 2019). IRE is associated with tissue ablation; therefore, a higher PEF intensity is required. On the contrary, RE or gene therapy focuses on transient permeabilization of cells; therefore, the required electric field strength is much lower. Depending on the tissue heterogeneity and electrical parameters, electroporation thresholds may vary. Nevertheless, numerical modeling could serve as a basis for treatment planning and selection of appropriate pulse parameters.

## 3 Electrodes

In this study, comparison of different electrode types was performed using FEM modeling in COMSOL Multiphysics, version 5.5 (COMSOL, Los Angeles, CA, United States). In order to simplify the calculations, each tumor was modeled as a three-dimensional homogeneous mass of tissue with conductivity 1.5 S/m and a relative permittivity of 80. Positive and zero potentials were set to corresponding electrode pairs depending on the electrode configuration. The electric potential value for each electrode configuration varied depending on the previously published protocols and is, therefore, reported along with the simulations. Outer boundaries of the geometry were treated as electrically insulated. Stationary analysis was performed to estimate the spatial distribution of the electric field.

### 3.1 Invasive electrodes

**FIGURE 3:**
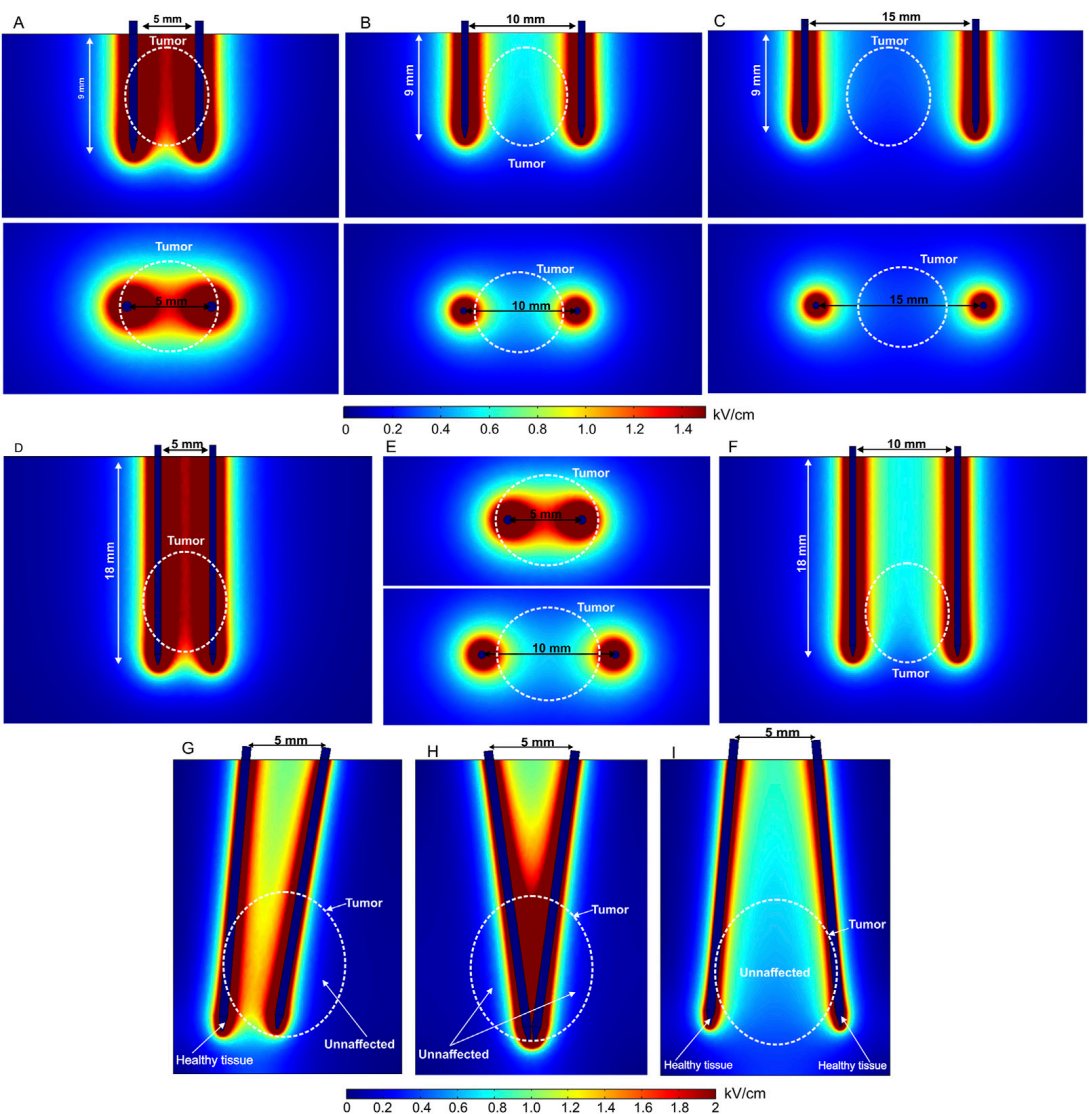
Spatial electric field distribution of two-needle fixed-position electrodes **(A–F)** and non-parallelism issue with adjustable electrodes **(G–I)** with different lengths and gap size, using 500 V terminal voltage: **(A)** 5 mm gap between electrodes; **(B)** 10 mm gap between electrodes; **(C)** 15 mm gap between electrodes; **(D)** 20 mm length electrodes with 5 mm gap; **(E)** 20 mm length electrodes with 5 mm and 10 mm gap, top view; **(F)** 20 mm length electrodes with 10 mm gap, side view; **(G)** electrodes are rotated by 10° and 5°; **(H)** both electrodes are rotated by 8° and −8°; **(I)** both electrodes are rotated by −10° and 10°.

Invasive electrodes require electrode penetration into a tissue. The electrode is usually needle-shaped, with a sharp tip. Therefore, most invasive electrodes deliver the electric pulses through typically stainless-steel needles of different length. Currently, fixed-position as well as adjustable position (electrodes, IGEA Medical; Electrodes for *in vivo* electroporation, 2021a) or needle composition (Adjustable Electrodes, 2022) electrode pairs or arrays are commercially available. Fixed-position electrodes are further categorized into two-needle electrodes (Isobe et al., 2004; Chen et al., 2015; Langus et al., 2016; Zager et al., 2016; Yao et al., 2017) and longitudinal or hexagonal electrode arrays. Basically, in this category, the electric field distribution is dependent on the number of needles (Adeyanju et al., 2012), length of the needles, gap spacing, and diameter of the needle tip (Davalos et al., 2005). The electric field distribution of the two-needle electrode configuration, including variation of gap size and length, is shown in Figures 3A–C.

Free position electrodes are advantageous when the tumor or tissue composition is not predetermined. Usually, this type of electrode is combined with an adjustable handle to fix the position after insertion into the target. Nevertheless, the needles are very thin; therefore, the distance between electrodes in deeper tissues may vary, which means the effects of non-parallelism should be considered since it affects the spatial electric field distribution (Figures 3G–I).

**FIGURE 4:**
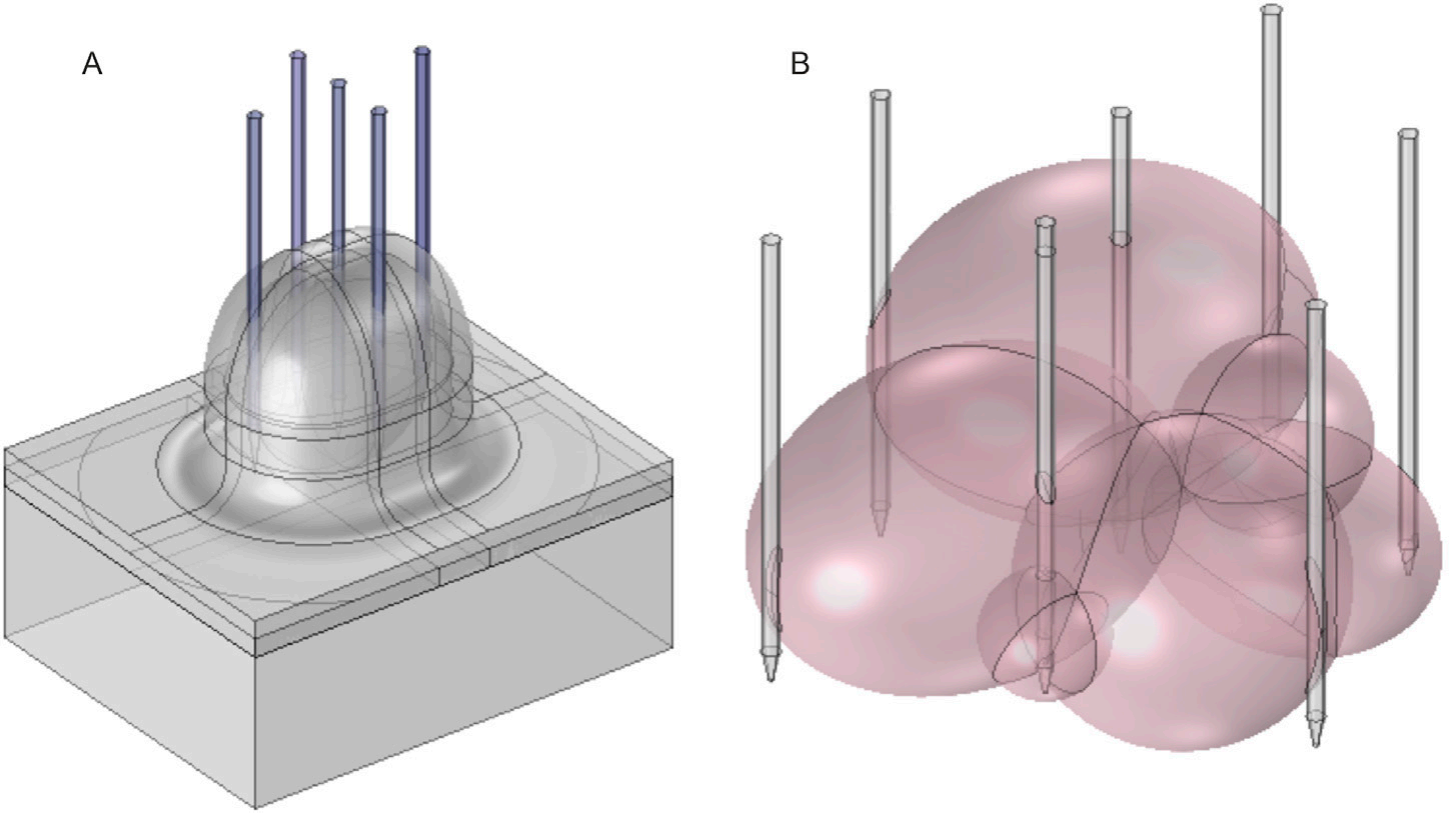
Array of adjustable needle electrodes for **(A)** corneous tumors and **(B)** deep-seated tumors.

As can be seen in the aforementioned figures, the visible gap size in each case is the same—5 mm; therefore, the top view does not change (Figures 3G–I). However, non-parallelism may occur in deeper tissues, especially when using longer needles. Typically, this occurs due to skin surface curvature and composition (Wei et al., 2017; Kopcewicz et al., 2020). Therefore, non-parallelism leads to electric field inhomogeneity and non-uniform treatment, as potentially healthy tissue is affected and target tissues (white dashed lines representing the tumor) remain unaffected or treated insufficiently. It is clear from Figure 3 that using two-needle electrode configurations may involve inhomogeneity of the PEF distribution. This limitation can be minimized using an array of adjustable needles as shown in Figure 4.

Needle arrays or repositioning of needles can be used to ensure more homogeneous deep-seated tumor electroporation. Placement of needle electrodes of variable geometry is adjusted to the individual size and shape of the tumor. To obtain an above-threshold electric field and cover the entire tumor volume, multiple needles are placed at the tumor margins and/or within the tumor. At the same time, the number of needles should be limited to reduce treatment invasiveness and complexity. The electrical pulses are, therefore, subsequently delivered between predetermined needle pairs. Such an electroporation procedure requires very precise pretreatment planning and PEF parameter evaluation (Miklavcic et al., 2010; Pavliha et al., 2012a; Blazevski et al., 2020). However, it enables efficient treatment of tumors with multiple nodules (Figure 4B). Nevertheless, non-parallelism is still a problem and is usually solved by x-ray imaging during the operation after electrode positioning and fixation (Moreta-Martínez et al., 2022). If non-parallelism is detected, adjustment of the treatment parameters and/or repositioning of the electrodes can be performed.

When the target tissue or tumor is subcutaneous, the electroporation procedure requires access to deep-seated cancer lesions without making a large incision in the skin. Such treatment is performed using an open laparoscopy approach or trans-oral and trans-anal endoscopy through a catheter (Lee et al., 2019; Li et al., 2021). Therefore, the requirements for the electrodes become more complex: electrodes have to be placed strictly parallel in order to ensure homogeneous PEF; the procedure must be performed on a relatively small probe area; and at the same time, the operating area has to cover the whole tumor volume.

**FIGURE 5:**
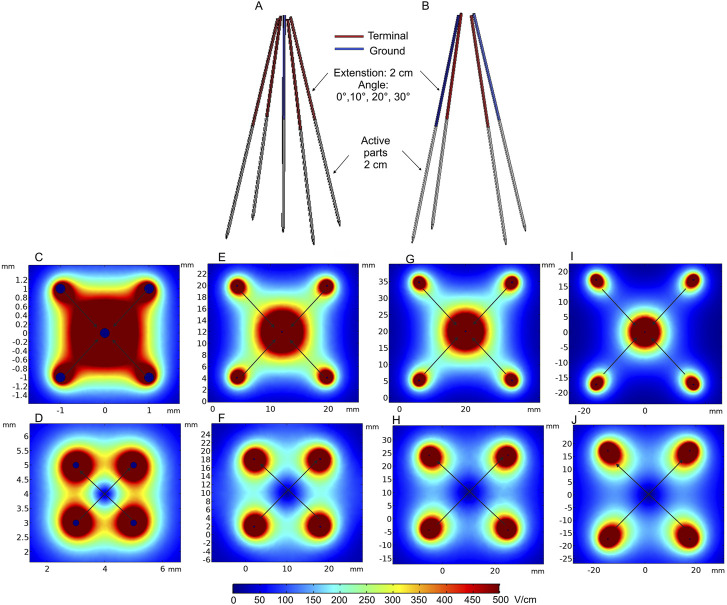
Deployable expandable electrodes: **(A)** 5-needle electrode structure; **(B)** 4-needle electrode structure with 2 cm extension; **(C)** spatial electric field distribution of the 5-needle electrode when positioned at 0° angle; **(D)** spatial electric field distribution of the 4-needle electrode when positioned at 0° angle; **(E)** spatial electric field distribution of the 5-needle electrode when positioned at 10° angle; **(F)** spatial electric field distribution of the 4-needle electrode when positioned at 10° angle; **(G)** spatial electric field distribution of the 5-needle electrode when positioned at 20° angle; **(H)** spatial electric field distribution of the 4-needle electrode when positioned at 20° angle; **(I)** spatial electric field distribution of the 5-needle electrode when positioned at 30° angle; **(J)** spatial electric field distribution of the 4-needle electrode when positioned at 30° angle. *Simulation performed includes single diagonal and semi-diagonal terminal voltages (0°—120 V, 10°—1100 V, and 20° and 30°—1700 V).

An electrode prototype considering the aforementioned features was presented by Izzo et al. (2020). The study shows the evaluation of the effectiveness and suitability of deployable and expandable 4-needle or 5-needle electrode configurations for IRE *via* laparoscopy and open surgery in the liver of a pig. The electrodes were also tested with trans-anal and trans-oral endoscopic approaches using different electrode configurations. All procedures were performed under ultrasound guidance. The authors state that the electrodes and their mechanical functionality are suitable for the listed procedures, and the electrodes are compatible with the 5-mm laparoscopic trocar and other surgical instruments. Laparoscopic and endoscopic approaches to deep-seated tumors could potentially minimize the risk of bleeding and infection. The FEM model of such an electrode array is presented in Figures 5A, B. Colored needle parts represent the non-conductive adjustable 2-, 3-, or 4-cm-length electrodes, positioned at 0°, 10°, 20°, and 30° angles. The deployable electrodes connected to high and ground potentials are shown in red and blue, respectively, with a fixed length of 2 cm.

The expected spatial electric field distribution at different insertion angles of the electrodes (0° 10°, 20°, and 30°) is shown in Figures 5C–J. The computations were performed with 2-cm needle extension. Diagonal and semi-diagonal black arrows represent the pairs of electrodes where the voltage is applied. It can be seen that by changing the active electrode pairs, the volume of the electroporated tissue can be controlled. If required, the whole volume could be ablated and/or reversibly electroporated due to overlapping of the high-intensity PEF regions. The capability to increase the length of each needle independently also allows for controlling the depth of the electroporated volume. Essentially, these electrodes are a specific case of the applicators presented in Figure 4, but when non-parallelism is intentional. The problems of precise needle positioning are still applicable.

**FIGURE 6:**
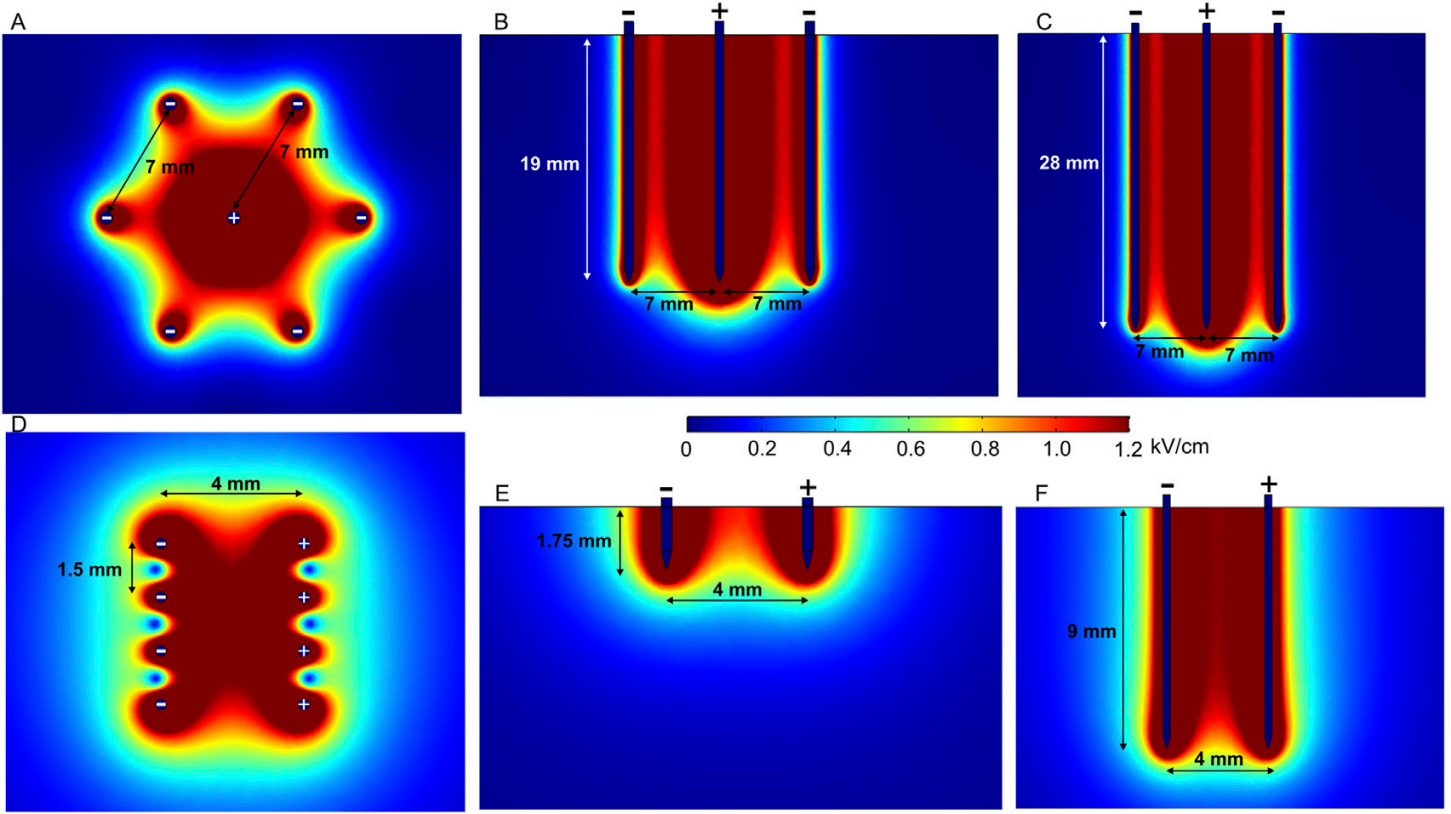
Spatial electric field distribution generated by hexagonal and intradermal needle-type electrode arrays with 1500 V and 600 V terminal voltages, respectively, taking into account different depths of penetration. **(A)** Hexagonal electrodes, top view; **(B)** hexagonal 20-mm-length electrodes, side view; **(C)** hexagonal 30-mm-length electrodes, side view; **(D)** intradermal electrodes, top view; **(E)** intradermal 2-mm-length electrodes, side view; **(F)** intradermal 10-mm-length electrodes, side view.

At the same time, fixed-position electrodes are advantageous for minimizing non-parallelism during needle insertion. Electric field distribution analysis of a hexagonal array of four-needle pair fixed electrodes is represented in Figures 6A–F. In the first case (Figures 6D–F), the applied voltage is 600 V, which induces a relatively homogeneous electric field between the positive and negative electrode pairs of 2- and 10-mm needle lengths.

Such electrodes are commercially available and used for intradermal (ID) electroporation, featuring 2- and 10-mm electrode lengths (Needle Array Electrodes for BTX AgilePulse *In Vivo*, 2021; Fixed Electrodes, 2022b). Intradermal electrodes are used when the penetration of outer skin layers, i.e., the stratum corneum, dermis, and epidermis, is sufficient. The spatial electric field distribution generated by an ID electrode with two rows of four needles is presented in Figures 6D–F. There is a notable difference in spatial electric field distribution in the tissue; 10-mm needles inserted at approximately 7 mm depth provide a uniform electric field; on the contrary, 2-mm electrodes feature a less homogeneous electric field distribution in the effective volume of effect. Nevertheless, when the limitations are taken into account, electrodes can be successfully utilized in practice (Roos et al., 2006; 2009; Lladser et al., 2010).

In the case of hexagonal electrodes (Figures 6A–C), the electric field is located around the positively charged electrode; therefore, the electric field is highest in the central part of the target tissue, while potentially healthy tissue on the edges remains intact. These electrodes are suitable for bigger tumors in the ECT context (Pichi et al., 2018), and different similar configurations can be used for gene therapy (Gilbert et al., 1997).

To summarize, fixed-position electrodes are likely to produce a more uniform electric field due to better control of tissue penetration—the chances of non-parallelism of electrodes are minimized. However, fixed-position electrodes are suitable only for tumors of predetermined size and, therefore, are less flexible for cancer treatment, especially when the tumor size is significantly smaller or bigger than the gap between the fixed electrodes. ID electrodes are appropriate for gene therapy; however, the penetration depth should be considered to ensure sufficient homogeneity of the electric field.

**FIGURE 7:**
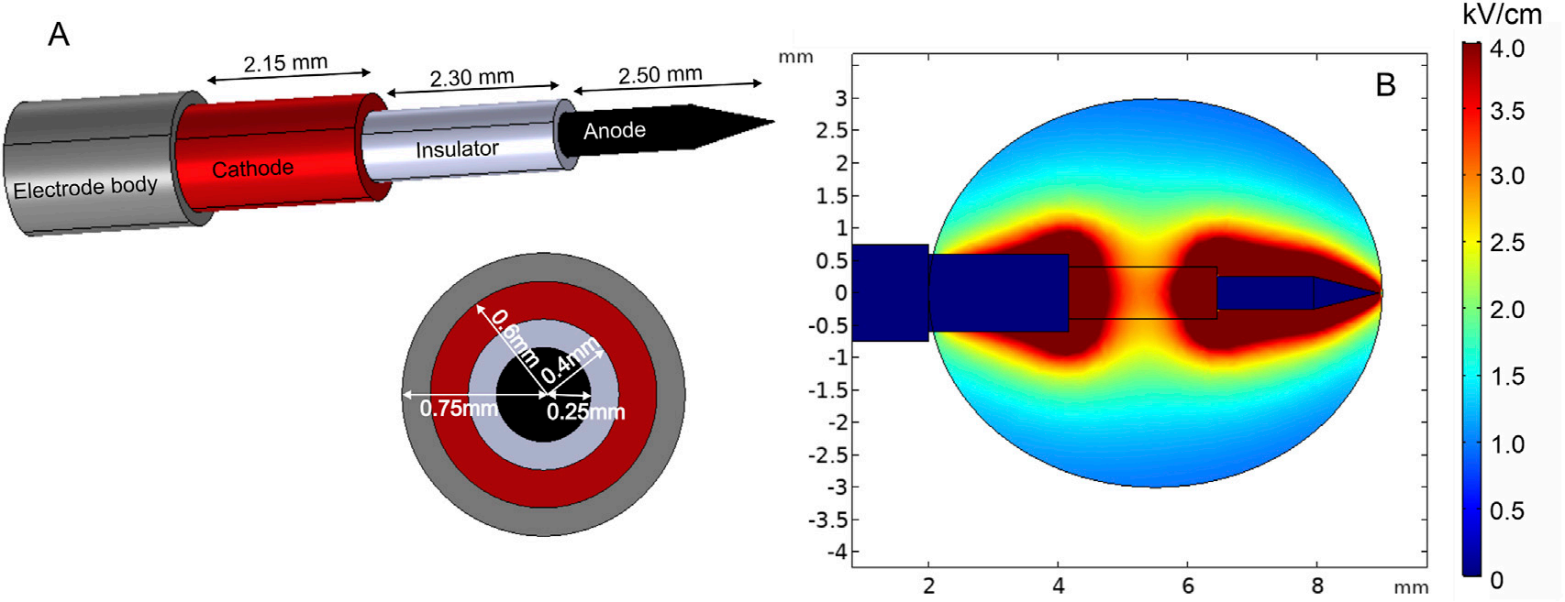
Single-needle electrode model: **(A)** electrode structure; **(B)** spatial electric field distribution with 1300 V terminal voltage.

One of the solutions to minimize the challenges of electrode positioning is the use of single-needle electrode configuration (Neal et al., 2010; Garcia et al., 2014). Such an electrode type consists of an electrode body, cathode, insulator, and anode on the sharp tip of the needle, where each part has a predetermined length and width (Figure 7A). Figure 7B shows the spatial electric field distribution. It can be seen that the design solves the problem of non-parallelism; however, as a trade-off, the electric field distribution is relatively non-homogeneous. Moreover, the diameter of this electrode is relatively large; therefore, it is applicable mainly for bigger tumors.

**FIGURE 8:**
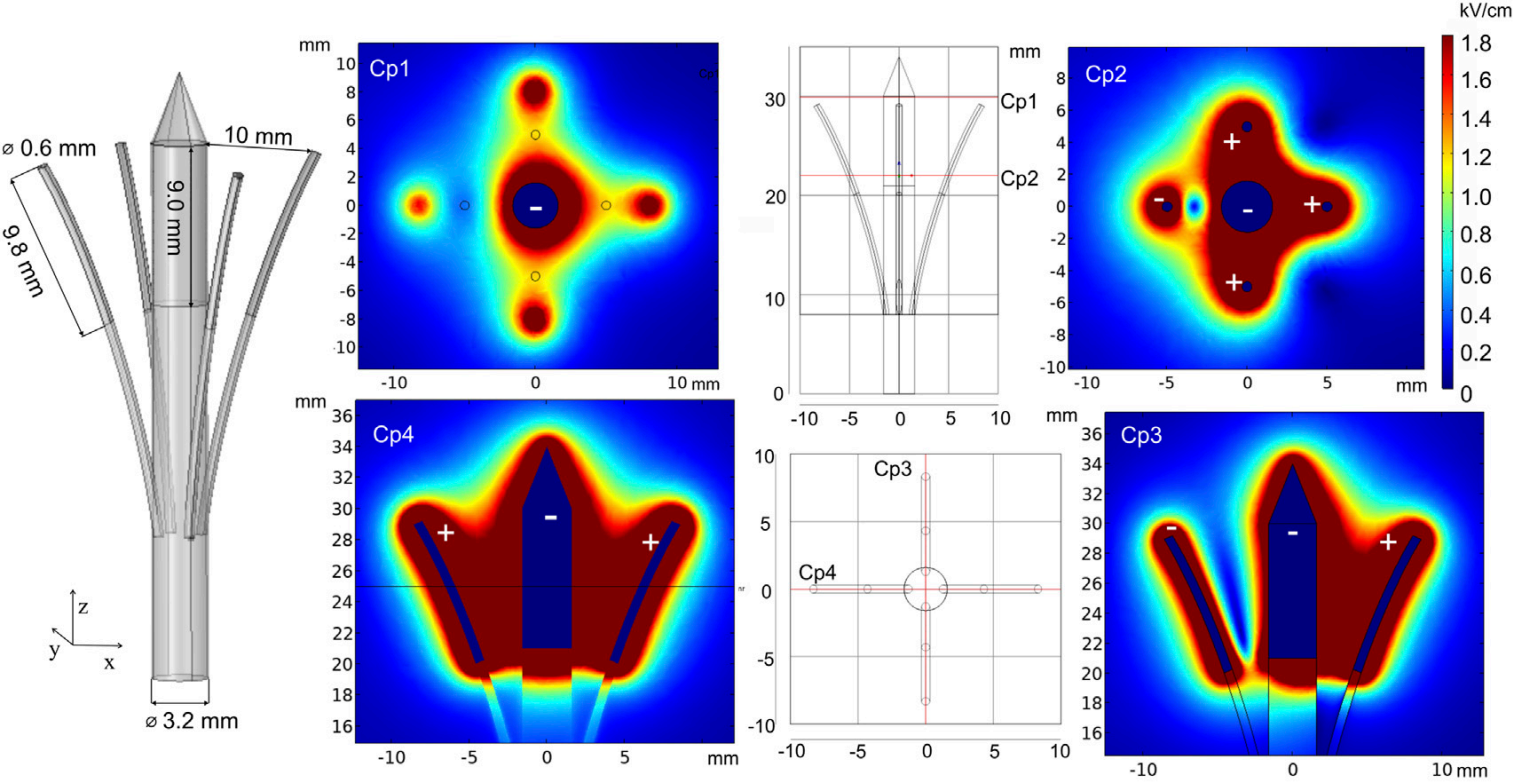
Curved electrodes using 1500 V terminal voltage. *Cp represents cut planes for electric field distribution analysis.

The new prototype of invasive electrodes, called curved electrodes, was proposed by Ritter et al. (2018). Curved electrodes are minimally invasive electrodes consisting of a penetrating central needle and four thin hollow expandable electrodes for pulse application and injection of chemotherapeutic agents. For simulation, the electrode terminal voltage was set to 1500 V. Furthermore, electric field distribution analysis was performed and is presented in Figure 8. Cut plane Cp1 shows the field strength on the surface when the penetration depth is shallow. As it can be seen, the highest electric field is around the central needle and at the positively charged satellite electrode tips. As a consequence, other areas will be treated with a lower PEF. However, penetration into deeper tissue layers results in a more homogeneous treatment (Cp2), which is also supported by the side view simulation (Cp3 and Cp4). Changing the number of active electrodes allows for controlling of the treatment volume. Moreover, the position of satellite needles can be adjusted with the movable part, which introduces additional flexibility.

To conclude, needle electrodes are advantageous for deep-seated tumors and intramuscular or intradermal GET targets. Both fixed-position and independent needle arrays show acceptable performance and are applied in clinical treatment. Fixed-position electrodes are easier to apply, but they are mostly suitable for specific size targets, i.e., the target should be of similar size as the gap distance. Otherwise, the healthy tissue will receive unnecessary pulsing. On the contrary, if the target exceeds the space between the electrodes, the treatment will result in partial response due to only a fraction of the tumor being affected. To overcome this problem, manual needle repositioning or more accurate multiple needle application along with a brachytherapy grid (van den Bos et al., 2016) may be considered. In addition, such fixing equipment and non-conductive ring nuts or “stoppers” help minimize non-parallelism after needle penetration. When the tumor is deep-seated, the requirements for treatment and electroporation electrodes are even more intricate; thus, tumor boundaries cannot be seen with the naked eye. Real-time imaging, such as ultrasound (van den Bos et al., 2016; Hsiao and Huang, 2017) or fluoroscopy (Pavliha et al., 2012b) guidance, is a solution. The combination of invasive electrodes with an imaging procedure gives the possibility for more accurate target boundary assessment, minimizing the possibility of multiple pulsing on the same area of the tissue since the field strength for each electrode pair is predetermined. Real-time imaging is also advantageous when tumors can be reached through the skin without incision (Lee et al., 2007).

### 3.2 Minimally invasive electrodes

**FIGURE 9:**
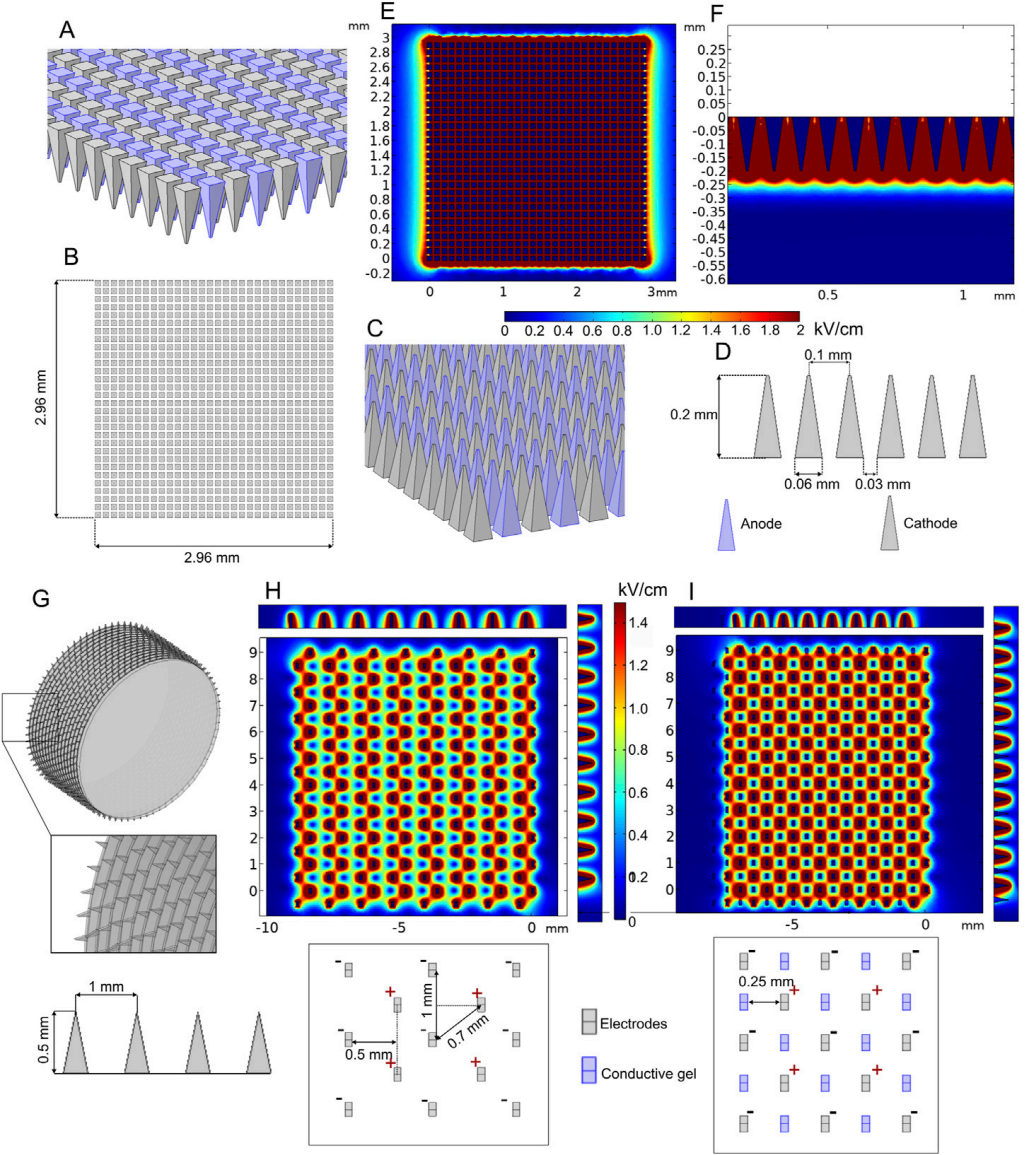
Microneedle array **(A–F)** and multi-needle roller **(G–I)** electrodes when 100 V voltage is applied: **(A–D)** microneedle array model; **(E)** spatial electric field distribution, top view; **(F)** spatial electric field distribution, side view; **(G)** multi-needle roller model; **(H)** spatial electric field distribution without conductive gel; **(I)** spatial electric field distribution with conductive gel channels.

Various minimally invasive (Choi et al., 2010; Yan et al., 2010; Xia et al., 2021) and non-invasive (Heller et al., 2010; Guo et al., 2011) microneedle array electrodes are mainly designed for transdermal drug delivery. The aim of microneedles is to affect the outer skin layers or muscle and ensure distribution of sufficient electric field for reversible electroporation, which is usually employed for electroporation-based gene delivery (also called gene vaccination). An example of such an array of electrodes is presented in Figures 9A–F.

The model consists of 900 (30 per row and 30 per column) 0.2-mm-length microneedles placed at 0.1 mm distance between needle tips. Terminal and ground potential electrodes are distributed in each row as shown in Figures 9A, C, and field analysis was performed using 100 V terminal voltage. The results are summarized in Figures 9E, F.

It can be seen that the depth of high-intensity electric field penetration is limited; however, it is still sufficient for transdermal gene delivery. The most significant disadvantage of most gene therapy electrodes is the relatively small operating area. In order to increase the effective area of the minimally invasive electrodes, roller type electrodes can be used as proposed by Yang et al. (2021). In Figure 9G, the model of such an electrode structure is shown, and the expected electric field distribution is presented in Figure 9H.

The FEM analysis results indicated that the highest value of electric field strength is located around the microneedle tips; however, it decreases drastically in the gap between the negatively and positively charged needle pairs (Figure 9H). A similar electrode type was analyzed by Huang et al. (2018). In order to improve the non-homogeneity, it was proposed to combine the structure with conductive gel microchannels, which are formed by applying gel on the skin and rolling the needles on the skin ten times before the pulsing. As an approximation, our simulation of this treatment covers the electric field distribution with one layer of microchannels filled with conductive gel (Figure 9I). The results indicated that the conductive gel channels can improve electric field homogeneity.

### 3.3 Non-invasive electrodes

As previously mentioned, non-invasive electrodes interact through the skin interface. Non-invasive electrodes are less suitable for deep subcutaneous tumors; however, they may be advantageous on exophytic tumors or melanoma, which appears on the skin surface. Several configurations of such electrodes are presented in the following.

**FIGURE 10:**
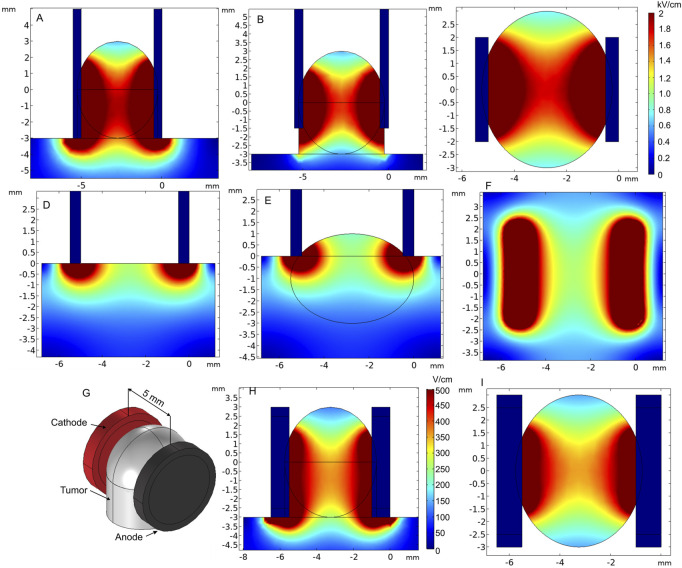
Spatial electric field distribution of plate and round tweezer electrodes using 1000 V and 200 V terminal voltages, respectively. **(A)** Electroporation of a superficial tumor, when plates embrace the tumor sufficiently, side view; **(B)** electroporation of a superficial tumor, when plates embrace the tumor insufficiently, side view; **(C)** electroporation of the superficial tumor, top view; **(D)** electroporation of the skin, side view; **(E)** electroporation of melanoma or a small superficial tumor; **(F)** electroporation of the skin or melanoma, top view; **(G)** round tweezer electrode simulation model; **(H)** spatial electric field distribution, side view; **(I)** spatial electric field distribution, top view.

Plate electrodes are most commonly used as a non-invasive electrode type (Al-Sakere et al., 2007; Sedlar et al., 2012; Novickij et al., 2021). The configuration consists of two rectangular stainless steel plates with fixed gap size [or adjustable with clippers (Caliper Electrodes for Electroporation Applications, 2022; Wang et al., 2008)] placed in parallel representing the anode and cathode (Fixed Electrodes, 2022a). Figures 10D-F show the application of plate electrodes for electroporation of skin (Figure 10D) and small skin lump (Figure 10E).

Tweezer-type electrodes are also a sub-population of parallel plate electrodes that are comfortable to be used when the gap between electrodes need to be adjustable. An example of round tweezer electrodes with adjustable 1–20 mm gap size is shown in Figures 10G–I (Tweezertrodes Electrodes for *In Vivo* and In Utero Electroporation Applications, 2022). For prediction of electric field distribution, we used a specific case with 5-mm-diameter and 4.5-mm-gap electrodes covering a tissue lump. As expected, due to limited contact area, the electric field was not homogeneous. Proper and maximum contact of the electrode surface with the tissue should be ensured for the electrodes to be applicable in cancer treatment. Nevertheless, the requirements for electric field homogeneity during gene therapy are lower; thus, this type of tweezer electrodes is sometimes favorable due to the ease of use and compactness (Maiorano and Mallamaci, 2009; Shi et al., 2010; Zhang et al., 2022). Tweezer electrodes are available in a variety of tip shapes and sizes (Tweezertrodes Electrodes for *In Vivo* and In Utero Electroporation Applications, 2022).

In the case of superficial tumors, good contact between the electrodes and the tissue is essential, as it may dramatically affect the electric field distribution (Figures 10A, B). Also, forming a lump can be sometimes advantageous. Nevertheless, it can be clearly seen that the top and bottom of the tumor are covered by a significantly lower electric field, especially when the tumor is embraced insufficiently (Figure 10B). If not taken into account during the treatment planning step, it may result in re-occurrence of the tumor.

**FIGURE 11:**
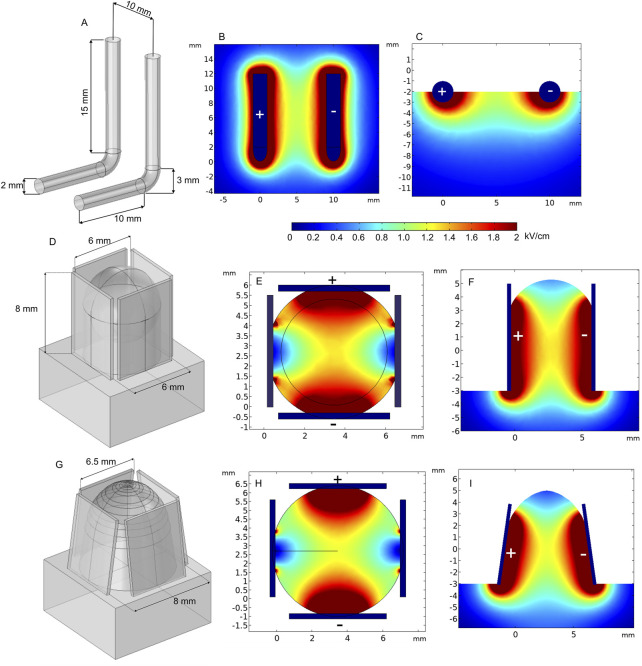
L-shaped **(A–B)** and 4-plate electrodes with **(D–F)** and without expansion **(G–I)** when 1300 V terminal voltage is used. **(A)** L-shaped electrode structure; **(B)** spatial electric field distribution, top view; **(C)** spatial electric field distribution, side view; **(D)** 4–plate electrode structure without plate expansion; **(E)** spatial electric field distribution without plate expansion, top view; **(F)** spatial electric field distribution without plate expansion, side view; **(G)** 4-plate electrode structure with plate expansion; **(H)** spatial electric field distribution with plate expansion by 8°, top view; **(I)** spatial electric field distribution without plate expansion by 8°, side view.

L-shaped electrodes are another commonly used applicator for electroporation-based treatments. This type of electrodes is mostly used for large cutaneous margins and is designed for the treatment of skin tumors of all sizes. An example of such a commercially available electrode arrangement is shown in Figure 11A (Accessories ElectroVet, 2021). For the simulation, the electrodes were placed on the tissue boundary and pushed into the skin; furthermore, 1300 V voltage was applied. Figures 11B, C show that the electric field of the L-shaped electrodes is inhomogeneous, i.e., the highest dose of PEF is expected at the skin surface, while deeper tissue layers are affected by a significantly lower electric field.

Similar L-shaped electrode configurations have been employed for *in vivo* electroporation for both gene therapy and electrochemotherapy by Mazères et al. (2008). In their study, electrodes were repositioned by 90° after each pulse train to compensate electric field inhomogeneity. The results showed 93% of complete response on treated animals, and the electrodes were efficient on tumors of up to 5 cm diameter. Gene therapy was also successful when performed using 4-mm-gap electrodes. However, ablation of the skin was observed.

Another option of non-invasive electrodes is the 4-plate electrode (4PE), where pulses are delivered by two parallel electrode pairs instead of rotating two plates by 90°. The 4PE was developed by Heller et al. initially for gene electro-transfer procedures (Heller et al., 2007). The electrodes operate as follows: metal plates are placed on the target to “grab” the desirable skin fold, and the non-conductive ring-shaped nut is tightened to establish a constant gap size. Two different gap sizes were analyzed—6 mm, without plate expansion, and 8 mm, with 8° expansion (Figures 11D,G, respectively). As can be seen from Figures 11E, F, the spatial electric field distribution of the 4PE is similar to that of two-plate electrodes. However, since the pulsing is performed between 90° shifted electrode pairs, during the second pulse train, the non-homogeneity of the treated volume can be better compensated.

According to the spatial electric field distribution presented in Figure 11F, the value of the electric field decreases at the top and the bottom of the electroporated tissue. The problem is even more apparent when the plates are expanded (Figure 11I). However, when the target tissue is larger in size, such expansion may be advantageous, which ensures a wider contact area by squeezing the tissue between all the plates.

**FIGURE 12:**
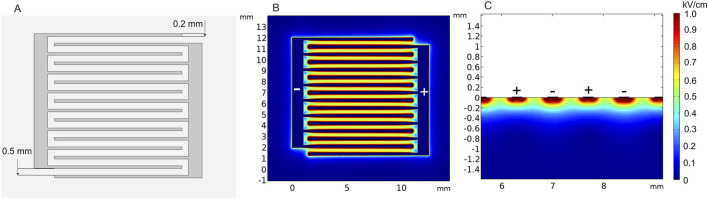
Micromachined pliable electroporation patch (ep-Patch) with rectanglular parallel gold electrodes, using 50 V terminal voltage. **(A)** Electrode structure; **(B)** spatial electric field distribution, top view; **(C)** spatial electric field distribution, side view.

Another type of superficial electrode currently being developed is the pliable electroporation patch with thin flexible electrodes. Currently, such electrodes are successfully employed for gene delivery. Flexible electrodes adapt to the skin surface and, therefore, ensure good contact. Figure 12 illustrates the micromachined pliable electroporation patch (ep-Patch), which consists of rectangular parallel gold electrodes presented by Wei et al. (2015). We analyzed a model with 0.2 mm width and 0.5 mm spacing between the electrodes (Figure 12A). The electric field distribution was evaluated with 50 V terminal voltage (Figures 12B, C). It can be seen that the electrodes ensure acceptable transdermal electric field distribution, while the flexibility to adapt to the skin surface is advantageous for practical applications.

As previously pointed out, electroporation success strongly depends on the electric field distribution in the tissue (Miklavcic et al., 2006). In order to guarantee effective treatment, homogeneous PEF must be spread through the whole target; otherwise, only partial treatment of the target will be achieved. This problem is especially apparent for non-invasive electrodes in ECT, resulting in tumor re-occurrence (Shankayi and Firoozabadi, 2012). Therefore, either alternative electrode configurations should be considered or a repositioning strategy developed during the treatment planning step.

Simulation results showed that non-invasive applicators are not capable of reaching subcutaneous or deeper tissues with sufficient PEF value. A higher pulse amplitude could be considered, but it would trigger IRE of the tumor in close proximity with the electrode terminals, which is not always desired. Increasing the amount of conductive gel between the electrodes and tissue fold improves the PEF distribution (Ivorra et al., 2008); however, the parts of the tumor without direct contact with the electrodes are likely to be affected by the insufficient electric field (Novickij et al., 2021). Additionally, too much conductive gel, especially in the top part of the tumor, can short-circuit the generator.

### 3.4 Partly invasive electrodes

Plate-and-fork-type electrodes are commercially available electrodes (Electrodes for *in vivo* electroporation, 2021b) that are effective for gene therapy (Maruyama et al., 2001).

**FIGURE 13:**
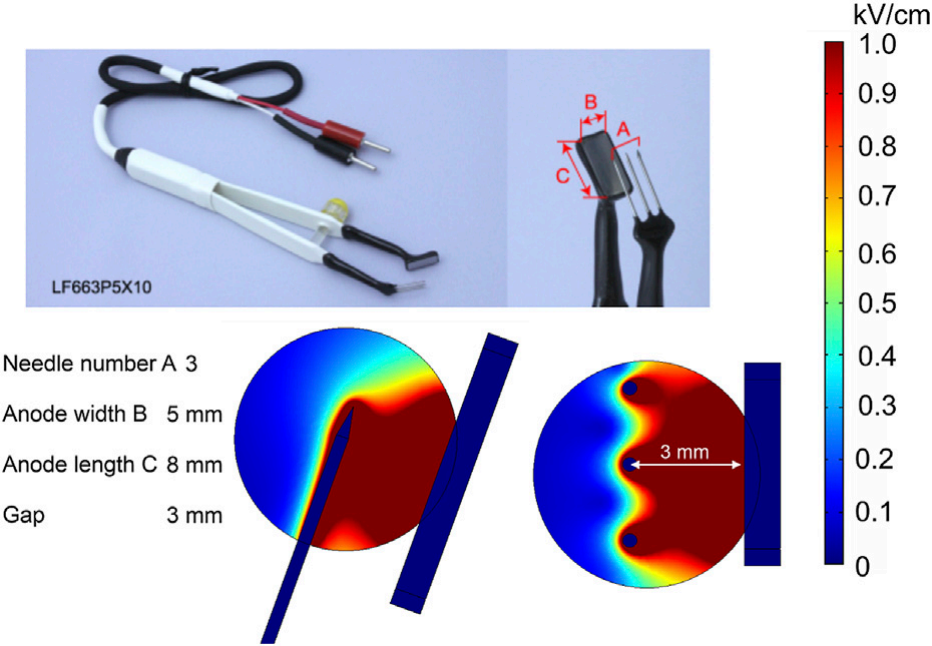
Plate-and-fork-type electrodes using 500 V terminal voltage. **(A)** Commercially available electrodes; **(B)** electrode structure; **(C)** spatial electric field distribution, side view; **(D)** spatial electric field distribution, top view.

The analyzed model consists of a pair of tweezers, one with a stainless steel fork consisting of 3-mm needles and the other with a 5 × 8-mm plate, and a spherical tumor (Figure 13). The fork with needles is inserted into the tissue, and simultaneously, the rectangular plate embraces the target. This electrode also contains a fixing part to keep the gap size stable between the fork and the plate while applying the pulses. Clearly, this is a convenient way for tight and accurate grasp of the target tissue; however, it does not solve the field homogeneity problem; thus, the application is limited to gene therapy.

## 4 Applications *in vivo* and in clinical trials

A variety of electrode prototypes and electrode geometries are commercially available; however, electrode structure is only one component of electroporation success. Optimal pulsing parameters need to be selected and adjusted according to the electrode structure and tissue properties to enhance treatment efficacy. In order to produce the aforementioned threshold PEF value, adequate pulse amplitude must be applied. Furthermore, modifications of other pulsing properties, i.e., duration, pulse shape, number of pulses, and repetition frequency, are considered and adapted to the electroporation protocol. The interest in pulsing parameter optimization is growing, following the possibility to manipulate the treatment outcome at the same time minimizing the side effects such as muscle contractions (Arena et al., 2011a), thermal damage (Mi et al., 2017), or pain sensation (Cvetkoska et al., 2022). Therefore, determination of appropriate pulsing protocol properties is an essential step in electroporation-based procedures. Currently, a wide range of PEF protocols have been introduced and applied in practice. We have summarized the pulsing protocols and applications of previously reported electrodes in Supplementary Table S2.

The presented limitations and advantages of each electrode structure can significantly determine the electroporation-based clinical treatments. For instance, skin cancer (melanoma, squamous or basal carcinoma, etc.) patients are typically cured via plate or needle array (parallel row or hexagonal) electrodes combined with the ECT procedure, which includes chemotherapeutic agents and ESOPE established electric pulses (Miklavčič et al., 2014). In order to overcome the presented non-homogeneity of the electric field, especially at the central part of the tissue, the electrodes are repositioned or slightly shifted sideward, and simultaneously, subsequent doses of PEF are applied (Campana et al., 2014). The strategy indeed has a positive influence on improving the electric field and, thus, diminishing the growth dynamics of cancerous cells, although it cannot guarantee a minimal ablation area. The necrotic skin areas that were in contact with the electrodes were reported to heal within a month (Quaglino et al., 2008) in most cases, followed by mild to severe pain (Kunte et al., 2017). The mentioned factors may cause discomfort for patients. Nonetheless, ECT may offer an effective treatment for cancerous skin lesions: basal cell carcinoma, 100% complete response within 15–56 months (Kis et al., 2019); melanoma metastases, 89% complete response within 24 months (Ricotti et al., 2014) and 60% complete response within 6 months (Matthiessen et al., 2011); and malignant melanoma, 53.5% (Ferioli et al., 2022). Partial or negative tumor response may potentially be associated with inappropriate drug concentration, non-uniform PEF distribution, and other factors, including immune response to the treatment (Sersa et al., 2008b; Cadossi et al., 2014).

ECT has also proved to be an efficient method in the treatment of deep-seated tumors. The procedure is usually performed with fixed or variable length, composition, and number of needle electrodes, which are injected with ultrasound guidance percutaneously or with open surgery (Gerlini et al., 2013; Djokic et al., 2020). However, ultrasound real-time monitoring alone may sometimes not be accurate enough for precise targeting of the target tissue (Eisele et al., 2014). Recently, laparoscopic approaches with ultrasound support for ECT have been introduced to facilitate electrode guidance for tissue penetration (Stillström et al., 2017; Izzo et al., 2020). Such procedures are advantageous in terms of lesser complications, faster procedure, and smoother patient recovery. The appropriate needle positioning strategy for each specific procedure is performed individually using computed tomography and/or magnetic resonance images prior to treatment (Djokic et al., 2018b). The placement, number of needles, and exposure activation plan are then selected in the most efficient manner using various techniques or software. One such technique was introduced by Marčan et al. The developed web-based electric field distribution visualization tool can be successfully adopted for accurate and time-efficient pre-treatment planning (Groselj et al., 2015; Marčan et al., 2015). Nevertheless, the complete response of deep-seated targets in different locations is still considerably lower than that of skin treatments: 55.5% in <1 month (Coletti et al., 2017), 63% within 20.2 months (Edhemovic et al., 2020), and 50% within 2 months (Matthiessen et al., 2018)), although, in most cases, chemotherapy or radiotherapy is performed before ECT.

IRE is another commonly used electroporation-based tumor ablation method (Aycock and Davalos, 2019). IRE procedures are traditionally performed using relatively long (10 µs–20 ms) monophasic pulses with 1 Hz pulse repetition frequency (Jiang et al., 2015). However, studies confirm that such electric pulsing protocols distinguish many negative factors, i.e., muscle contractions and thermal damage. In recent years, the novel modality of bipolar high-frequency pulses for non-thermal IRE treatment, termed H-FIRE, was proposed (Arena et al., 2011b). The H-FIRE procedure with adjustable position needle electrodes has been recently employed for prostate cancer. The results showed good tumor response to treatment and reduced muscle contractions during the procedure (Dong et al., 2018).

Gene electro-transfer procedures focus on the delivery of DNA encoding therapeutic transgenes mainly for cancer-related therapies or infectious disease vaccines (Heller and Heller, 2015), which addresses the activation of immune response to the treatment (Cervia and Yuan, 2018). Currently, these methods are under investigation in *in vitro* or animal models (Milevoj et al., 2019; Brezar et al., 2020). So far, the procedure was employed only in several clinical treatments, including melanoma (NCT00323206) with interleukin-12 plasmid (Daud et al., 2008), malignant tumors with AMEP plasmid (NCT01664273) (terminated), metastatic melanoma with AMEP plasmid (NCT01045915) (Spanggaard et al., 2013), and cutaneous basal cell carcinoma located in the head and neck region with phIL12 plasmid (NCT05077033) (Groselj et al., 2022). Depending on the target tissue, invasive (needles or needle arrays), minimally invasive (microneedle array or microneedle roller), or non-invasive (plate, patch, etc.) electrodes are selected to achieve maximum GET efficiency. Interestingly, it was found that moderate tissue preheating before pulse exposure could potentially enhance gene expression while reducing the PEF strength. The minimally invasive electrode array (MEA) with optical fibers for heat production was introduced by the Heller group (Edelblute et al., 2021). The purposed technique was applied on the skin; however, gene expression was also present in deeper layers, including the muscle (Bulysheva et al., 2019). DNA vaccines are another promising field of GET application; however, they still require improvement before clinical applications (Gothelf and Gehl, 2012; Cao et al., 2022).

## 5 Conclusion

Electroporation effectiveness varies depending on PEF spatial distribution in the tissue. Therefore, research and development of optimal pulsing protocols and applicators for electrochemotherapy (ECT), gene therapy (GT), or irreversible electroporation (IRE) is constantly performed. Currently, there are many types of electrodes (invasive, non-invasive, or minimally invasive); however, all of them have a niche for application and a universal structure is yet to be proposed. The current state-of-the-art is to compensate the problems of tissue heterogeneity and field inhomogeneity with real-time imaging during the procedure. Additionally, treatment planning steps may include FEM simulation of spatial electric field distribution and possible thermal effects.
