# Supplementary material for: Invasive and non-invasive electrodes for successful drug and gene delivery in electroporation-based treatments
Source: Front Bioeng Biotechnol. 2023 Jan 16;10:1094968. doi: 10.3389/fbioe.2022.1094968 (PMC9885012; doi:10.3389/fbioe.2022.1094968)
Supplement: Supplementary file 1 [file Table1.DOCX]

Supplementary Material

| TABLE I  Tissue electrical properties | | | | | | | | | |
| --- | --- | --- | --- | --- | --- | --- | --- | --- | --- |
| Tissue | σ LF,  Sm^-1^ | References | σ HF,  Sm^-1^ | References | ε_r_, LF, | References | ε_r_, HF, | References |  |
| Skin (dry) | 0.00565–0.25  0.0009  0.090–0.017  0.07 | (Low Frequency (Conductivity) » IT’IS Foundation)  (Gabriel et al., 1996)  (Wake et al., 2016)  (Hershkovich et al., 2019) | 38.18 | (Arab et al., 2020) | 1111  5340 | (Gabriel et al., 1996)  (Hershkovich et al., 2019) | 6.565 | (Arab et al., 2020) |  |
| Skin (wet) | 0.059 | (Gabriel et al., 1996) | 42.97 | (Arab et al., 2020) | 1136 | (Gabriel et al., 1996) | 8.537 | (Arab et al., 2020) |  |
| Stratum corneum | 0.0002–0.002 | (de Santis et al., 2015) |  |  | 8000–700  1000000–50000 | (Birgersson et al., 2011)  (Yamamoto and Yamamoto, 1976) | 700–400  50000–100 | (Birgersson et al., 2011)  (Yamamoto and Yamamoto, 1976) |  |
| Epidermis | 0.018 ± 0.008 | (Wake et al., 2016) | ~0.35 | (Wake et al., 2016) | 100000–62500 | (Tsai et al., 2019) | 62500–50000 | (Tsai et al., 2019) |  |
| Dermis | 0.434 ± 0.056 | (Wake et al., 2016) | ~0.45 | (Wake et al., 2016) | 1000000–75000 | (Tsai et al., 2019) | 75000–50000 | (Tsai et al., 2019) |  |
|  |  |  |  |  |  |  |  |  |  |
| Subcutaneous fat | 0.024–0.215  0.147 ± 0.020  ~0.2 | (Low Frequency (Conductivity) » IT’IS Foundation)  (Wake et al., 2016)  (Hershkovich et al., 2019) | ~0.15  ~0.22 | (Wake et al., 2016)  (Hershkovich et al., 2019) | 8648–617 | (Gun et al., 2017) | 175–58 | (Gun et al., 2017) |  |
|  | ~0.1 | (Gabriel et al., 2009) | ~0.1 | (Gabriel et al., 2009) |  |  |  |  |  |
| Muscle | 0.1–0.726 | (Low Frequency (Conductivity) » IT’IS Foundation) |  |  |  |  |  |  |  |
| *longitudinal* | ~0.37 | (Ahad et al., 2010) | ~0.64 | (Ahad et al., 2010) | ~71167 | (Ahad et al., 2010) | ~8700 | (Ahad et al., 2010) |  |
|  | ~0.4 | (Nagy et al., 2019) | ~0.6 | (Nagy et al., 2019) | ~92000 | (Nagy et al., 2019) | ~9500 | (Nagy et al., 2019) |  |
| *transverse* | ~0.15 | (Ahad et al., 2010) | ~0.46 | (Ahad et al., 2010) | ~53467 | (Ahad et al., 2010) | ~11867 | (Ahad et al., 2010) |  |
|  | ~0.21 | (Nagy et al., 2019) | ~0.42 | (Nagy et al., 2019) | ~87500 | (Nagy et al., 2019) | ~40230 | (Nagy et al., 2019) |  |
| Tumor | 0.22–0.40  0.00135 ± 0.19  0.166–0.222  0.411–0.461 | (Miklavčič et al., 2006)  (Ivorra et al., 2009)  (Laufer et al., 2010)  (Haemmerich et al., 2009) | 0.279–2.346  0.246–0.272  0.504–0.533 | (Cheng and Fu, 2018)  (Laufer et al., 2010)  (Haemmerich et al., 2009) | 99000–8600 | (Laufer et al., 2010) | 24.842–15.12  5100–3000 | (Cheng and Fu, 2018)  (Laufer et al., 2010) |  |
| Liver | 0.0636–0.43  0.03–0.091  0.075–0.179 | (Low Frequency (Conductivity) » IT’IS Foundation)  (Laufer et al., 2010)  (Haemmerich et al., 2009) | 0.124–0.164  0.260–0.288  ~0.2 | (Laufer et al., 2010)  (Gun et al., 2017)  (Gabriel et al., 2009) | 82000–11000  82306–18899 | (Laufer et al., 2010)  (Gun et al., 2017) | 6500–3400  1833–262 | (Laufer et al., 2010)  (Gun et al., 2017) |  |
|  | ~0.075 | (Gabriel et al., 2009) |  |  |  |  |  |  |  |
| Melanoma | 0.00000265 | (Glickman et al., 2003) | 53.445 | (Arab et al., 2020) |  |  | 9.631 | (Arab et al., 2020) |  |
| Prostate | 0.436–0.743  0.411–0783  0.238–0.901 | (Low Frequency (Conductivity) » IT’IS Foundation)  (Neal et al., 2014)  (Santamaría et al., 2007) | 0.916–1.06  0.290–1.149 | (Neal et al., 2014)  (Santamaría et al., 2007) | 90200–449000 | (Santamaría et al., 2007) | 13000–1160 | (Santamaría et al., 2007) |  |
| Lung | 0.058–0.249  0.05–0.25 | (Low Frequency (Conductivity) » IT’IS Foundation)  (Wang et al., 2014) | 0.151–0.309  0.16–0.57 | (Yamazaki et al., 2013)  (Wang et al., 2014) | ~1000000–~10000 | (Wang et al., 2014) | 10000–100 | (Wang et al., 2014) |  |
| *inflated* | ~0.03 | (Gabriel et al., 2009) | ~0.06 | (Gabriel et al., 2009) |  |  |  |  |  |
| *deflated* | ~0.12 | (Gabriel et al., 2009) | ~0.2 | (Gabriel et al., 2009) |  |  |  |  |  |

LF – up to 100 kHz frequency

HF – 100 kHz and greater frequency
