# Supplementary material for: Invasive and non-invasive electrodes for successful drug and gene delivery in electroporation-based treatments
Source: Front Bioeng Biotechnol. 2023 Jan 16;10:1094968. doi: 10.3389/fbioe.2022.1094968 (PMC9885012; doi:10.3389/fbioe.2022.1094968)
Supplement: Supplementary file 2 [file Table2.docx]

Supplementary Material

| TABLE II  ELECTRODES APPLICATION FOR *IN VIVO/EX VIVO* | | | | |
| --- | --- | --- | --- | --- |
| Electrode | Tumor/target type | Protocols | EP type | Ref. |
| **Invasive** | | | | |
| Needle pair | Sprague-Dawley rats myocardial decellularization | *Gap 10 mm*:  50 V, 100 µs, 1 Hz, 10p  250 V, 100 µs, 1 Hz, 10p  500 V, 100 µs, 1 Hz, 10p (significant)  500 V, 70 µs, 1, 2 or 4 Hz, 10p and 20p | IRE | (Zager et al., 2016) |
|  | Porcine livers | 3000 V, 70 µs, 20 A, 90p | IRE | (Chen et al., 2015) |
|  | Beef liver tissue (*ex vivo*) | *Gap 10 mm*:  500 V, 100 and 1000 µs, 1 and 4762 Hz, 8p  750 V, 100 µs, 1 and 4762 Hz, 8p  1000 V, 100 and 1000 µs, 1 and 4762 Hz, 8p | IRE | (Langus et al., 2016) |
|  | C3H strain mice Dunn murine osteosarcoma | 75 ms, 100 V/cm; 8p, 8 Hz | ECT | (Isobe et al., 2004) |
|  | Lung cancer and colorectal cancer | 1300 V/cm, 100 µs, 8p, 1 Hz | ECT | (Tremble et al., 2019) |
| Needle array | Feline nasal planum squamous cell carcinoma | 1000 V/cm, 100 µs, 8p, 5 kHz | ECT | (Tellado et al., 2022) |
|  | Female C57Bl/6 or Balb/c mice skin | *Gap 4 mm*:  100 µs, 1750 V/cm, 6p, ~8 Hz  100 µs, 200 V/cm, 6p, ~8 Hz  50 µs, 1125 V/cm, 2p, ~3.3 Hz  10 ms, 275 V/cm, 8p, ~3.2 Hz (significant)  50 µs, 1125 V/cm, 2p, ~2 Hz + 10 ms, 275 V/cm, 8p, ~1.96 Hz (significant) | GT | (Roos et al., 2006) |
| Needle array with reposition | Mice spine (Sp2/0) | ESOPE (1.4 kV/cm x 100 µs x 8);  800 ns, 3.5 kV/cm, 250 | ECT | (Novickij et al., 2020) |
|  | Prostate | 70–100 µs, 1500 V/cm, 90p | IRE | (Blazevski et al., 2020) |
| Curved | Liver (*ex vivo*) | 70 pulses, 100 µs, 1 Hz, 1.5 kV | IRE | (Ritter et al., 2018) |
| Intradermal | C57BL/6 mice melanoma | 50 µs, 1125 V/cm, 2p; 10 ms, 275 V/cm, 8p | GT | (Roos et al., 2009) |
|  | Human metastatic melanoma | 1250 V/cm, 100 µs, 1p + [1000 ms delay] + 140 V/cm, 400 ms, 1p | GT | (Spanggaard et al., 2013) |
| Hexagonal shape | Human head and neck malignancies | ESOPE | ECT | (Pichi et al., 2018) |
| Single needle | Female Nu/Nu mice (Crl:NU-Foxn1Nu) human breast carcinoma cells | 100 µs (4 sets changing polarity each 25p),  1300 V, 3 s intervals | IRE | (Neal et al., 2010) |
|  | Pancreas | 2250 V, (1-5-1, 2-5-2, and 5-5-5 μs) (energized time 100 μs, 300p) H-FIRE | IRE | (O’Brien et al., 2019) |
|  | Liver | 2250 V, 2-5-2 μs, (energized time 100 μs) H-FIRE | IRE | (Partridge et al., 2020) |
| Deployable expandable | Female Sus Scrofa liver | 100 µs x 5 kHz | IRE | (Izzo et al., 2020) |
| *5 needles* |  | *0°:* V_side_ 186 V + V_d/2_ 118 V x 80p  *20°, 20 mm:* V_side_ 1200 V + V_d/2_ 900 V x 120p  *20°, 30 mm:* V_side_ 1700 V + V_d/2_ 1100 V x 120p |  |  |
| *4 needles* |  | *10°, 20 mm:* V_side_ 1100 V + V_d/2_ 1700 V x 80p  *10°, 30 mm:* V_side_ 1500 V + V_d/2_ 2200 V x 80p  *20°, 40 mm:* V_side_ 1900 V + V_d/2_ 2700 V x 80p |  |  |
| **Minimally invasive** | | | | |
| Micro needle array | Human skin (hand) | 53, 108, 173 V;  1p – τ = 0.5 ms and τ = 1 ms;  3p – τ = 0.5 ms, 20 s between pulses | GT | (Choi et al., 2010) |
|  | Rat skin | 200 V, 10 ms, 10p, | GT | (Yan et al., 2010) |
|  | Porcine skin (*ex vivo)* | 296±25 V and -313±20 V (bipolar) ~10 µs (oscillations up to ~60 µs)  32.1±0.2 V, 52.2±4.4 ms or  99±5 V, 50.1±2.7ms (unipolar) | GT  DNA vaccines | (Xia et al., 2021) |
| Multi needle roller | C57BL/6 mice and BALB/c mice skin | 50 V, 10 ms, 1 s interval, 10p | GT | (Yang et al., 2021) |
|  | C57BL/6 mice skin | 50 V or 70 V, 10 ms, 1 s interval, 10p | GT | (Huang et al., 2018) |
| **Non-invasive** | | | | |
| Micro needle array | Guinea pig | 150 ms, 250 V/cm, 150ms delay, 72p (trough each pair) | GT | (Guo et al., 2011) |
| L-shaped | Horse sarcoid (ECT)  Mice skin surface (GT) | *Gap 9 mm:* 100 µs, 1170 V, 8x2p, 1Hz  *Gap 6 mm:* 20 ms, 60–240 V, 8p bipolar, 1Hz | ECT  GT | (Mazères et al., 2008) |
| 4–plate | C57/Bl6 mice skin surface | 25–800 V/cm, 1–200 ms, 1 Hz, 4+4p;  700–2000 V/cm, 10–1000 ms, 1 Hz, 4+4p | GT | (Heller et al., 2007) |
| Round tweezers | Mice uterus | *Gap 7 mm:* 50ms, 40 mV, 1 Hz, 4p with 950 ms intervals | GT | (Maiorano & Mallamaci, 2009) |
|  | Male mice testis | 40 V, τ=0.05 s, interval 1s. 8p (bipolar) | GT | (Shi et al., 2010) |
|  | Mice embryos | *Gap 2 mm:* 50 ms, 36 and 39 mV, 5p with  950 ms intervals | GT | (Zhang et al., 2022) |
| Plate | C57Bl/ 6 female mice LPB cell line | *Gap ~4 mm:*  100 µs, 2500 V/cm, 80p, 0.3 Hz | IRE | (Al-Sakere et al., 2007) |
|  | Mice SA-1 tumor | 100 µs, 1300 V/cm, (2 sets changing polarity each 4p), 1 Hz | ECT | (Sedlar et al., 2012) |
|  | Mice SA-1 muscle and IL-12 gen | 100 µs, 600 V/cm, 1p, 1 Hz + [1s delay] +  100 ms, 80 V/cm, 4p, 1 Hz | GT | (Sedlar et al., 2012) |
|  | C57BL/6 mice LLC1 tumor (on spine) | 1.4 kV/cm x 100 µs x 8  700 ns, 3.5 kV/cm, 1 MHz 200p  700 ns, 3.5 kV/cm, 1 kHz 200p | ECT | (Novickij et al., 2021) |
| Clipper | Rabbit skin  Mice skin | 100 V, 60ms and 60 Hz  50 V, 60ms and 60 Hz | GT | (Wang et al., 2008) |
| Planar ePatch | BALB/c nude mice skin | 20 ms, 40 V, 5p, 2s delay | GT | (Wei et al., 2015) |
| **Other** | | | | |
| Plate-and- fork-type | mouse/rat/human skin | 50 ms, 18 V, 1 Hz, 4p + 4 inversed | GT | (Maruyama et al., 2001) |
